# ‘The hardest job I’ve ever done’: a qualitative exploration of the factors affecting junior doctors’ mental health and well-being during medical training in Australia

**DOI:** 10.1186/s12913-021-07381-5

**Published:** 2021-12-14

**Authors:** Katherine Petrie, Mark Deady, Deborah Lupton, Joanna Crawford, Katherine M. Boydell, Samuel B. Harvey

**Affiliations:** 1 School of Psychiatry, University of New South Wales, Sydney, NSW 2000 Australia; 2 Black Dog Institute, Hospital Rd, Randwick, NSW 2031 Australia; 3 Vitalities Lab, Centre for Social Research in Health and Social Policy Research Centre, University of New South Wales, Sydney, NSW 2000 Australia

**Keywords:** Qualitative research, Mental health, Junior doctors, Medical training, Emotional labour, Professional hierarchies, Work stress, Occupational support, Transition

## Abstract

**Background:**

Medical practitioners can experience considerable stress and poor mental health during their careers, with doctors in training known to be particularly vulnerable. Previous research has documented work-related factors that may play a role in the mental health status of junior doctors. However, these and additional factors, need to be explored further by considering theory-driven, social, structural and contextual issues. This qualitative study aimed to explore the experiences of junior doctors working in Australian hospitals to identify factors that impact their mental health during medical training.

**Method:**

Semi-structured interviews were conducted with 12 junior medical officers (JMOs) employed across six hospitals in Australia. Transcribed de-identified interviews were analysed thematically using a data-driven inductive approach.

**Results:**

Four interrelated main themes were identified: i) professional hierarchies; ii) occupational stress; iii) emotional labour, and iv) taking distress home; which detail the complex affective, relational and professional experiences of JMOs. The accounts demonstrate how the social, professional and organisational dimensions of these experiences impact upon trainee’s well-being and mental health, both positively and negatively. Together, the findings document the dynamic, nuanced aspects of junior doctors’ experiences of medical training and practice and highlights the importance of relational connections and the workplace environment in shaping JMOs’ social and emotional well-being.

**Conclusion:**

The current study adds to the understanding of how junior doctors navigate medical training in Australian hospitals and highlights the complexities of this experience, particularly the ways in which mental health and well-being are shaped by different elements. These findings have important implications to inform new strategies to improve JMO mental health and to leverage work and non-work contexts to better support JMOs during medical training.

**Supplementary Information:**

The online version contains supplementary material available at 10.1186/s12913-021-07381-5.

## Background

Practising as a medical doctor can be a highly rewarding career. However, medical training and practice can be very demanding and adversely affect doctors’ mental and physical well-being [1–5]. There is now an extensive literature on the relationship between junior doctors’ working and training conditions and their mental health [3, 4], much of which is generated within a medical, psychological and quantitative framework. Internationally, a body of work using sociologically grounded analysis and critical qualitative inquiry has also explored junior doctors’ mental health and working conditions in greater detail [6–9].

Previous qualitative studies have investigated the social dimensions of experiences of the workplace and professional practice for medical practitioners, highlighting the process of professional socialisation [10] and supervision and teamwork [11, 12]. Qualitative studies with British and Australian junior doctors have revealed that much of the stress experienced as they transition from being a student to a working doctor is related to managing uncertainty [13, 14], developing competence [15], fear of making mistakes [6], feeling unsupported by senior colleagues [16], bullying and traineeship demands of exams and study [9]. However, fewer studies have addressed what they term ‘emotionality’ and the relationship between power and emotion [15].

Building on Hochschild’s [17] ground-breaking work on emotional labour, recent research on emotion rules and emotional labour in health care settings generally [18–20] has identified how feelings of stress, frustration, irritation or other distress are expected to be hidden or tightly controlled by health care professionals in an effort to appear competent, calm, empathetic and confident. This research is critical in highlighting how elements within and outside of the workplace can coalesce to generate different affective states among health professionals, including feelings of well-being or distress. This study extends this work by exploring these issues specifically among junior doctors undertaking postgraduate medical training. In doing so, we adopt a care-focused approach which recognises that junior doctors (henceforth referred to with the locally used title Junior Medical Officers [JMOs]) are not only caregivers but sometimes can also require care themselves to protect their mental well-being as they navigate a highly stressful period in their professional lives.

To date, Australia has been under-represented in this existing Anglophone literature, which is largely based in the USA, UK and Europe. To develop targeted strategies to address mental ill-health among junior doctors, it is important to understand the firsthand experiences of these issues within a given context, and qualitative methods are ideally suited to provide a detailed contextual understanding of how and why mental health problems arise within JMOs. Through semi-structured interviews, this study sought to illuminate the aspects of the work and training experiences of Australian JMOs and the implications for their mental health and well-being from the perspectives of the JMOs. The study adds to the limited qualitative Australian-based research and provides a comparative perspective to studies conducted in other wealthy countries. The study will inform future strategies to support and improve the mental health of JMOs. Specifically, this study aimed to undertake an in-depth qualitative exploration of the factors affecting junior doctors’ mental health and well-being during medical training in Australia.

## Method

Our study formed part of a larger multi-phase project to develop and evaluate a smartphone app to support the mental health of JMOs in Australian health care settings. The objective of this qualitative component was to inform the design and development of clinical content and to ensure the app was tailored to this target population. Semi-structured interviews followed a guide of open-ended questions with specific prompts, covering two topics: i) JMO life and well-being and ii) digital technology and mental health in general as well as within the JMO context. This article focuses on interviewees’ responses to the first topic. The topic guide is provided as Additional file 1**.** The project was granted full ethical approval by the South Eastern Sydney Local Health District Human Research Ethics Committee (HREC reference #: 18/140 (HREC/18/POWH/321).

Participants were recruited from hospital sites in Sydney between July and September 2018 via email invitations distributed by JMO managers, advertising through email and social media of three state professional organisations related to junior doctors, and on-site visits by members of the research team to JMO meetings and hospital Grand Rounds. A total of 41 JMOs initially expressed interest in the study. Of these, 12 provided written informed consent and participated in a face-to-face interview at a time convenient to them.

All interviews were audio-recorded and undertaken by a registered psychologist (a study collaborator and co-author [JC]). Interviews averaged 60 min and 32 s in duration. An external transcription agency provided de-identified verbatim transcripts of all interviews, which formed the basis of our analysis. To protect participants’ anonymity, gender-neutral pseudonyms and pronouns are used when reporting results. Circumstantial details were altered where required in cited quotations, and neither gender, real name nor age are reported to maintain privacy.

We undertook a thematic analysis in line with a similar study [21] based on a data-driven inductive approach, following Braun and Clarke’s six-stage process [22]. The developing analysis was led by the first author and then discussed with co-authors in an iterative process to refine ideas, discuss and develop consensus on a final set of themes. De-identified quotations from the interviews are provided to support our analysis.

## Results

### Participant characteristics

Participants included nine women and three men, aged 24–35 years of age (mean age = 28.9 years), employed across six hospitals. The participant group was spread across the early stages of medical training, comprising three interns (in initial stage of clinical training following graduation), five residents (with between one to 2 years of training) and four registrars (a position held after at least 3 years of clinical training). A range of specialties were represented, when disclosed. The majority of participants had received their medical degree at an Australian university. Over three-quarters had undertaken rural and or regional placements as part of their training. During the course of the interviews, four participants self-disclosed that they had sought professional help for mental health problems.

### Summary of themes

There were four main themes identified across the interviews, namely: i) professional hierarchies; ii) workplace stress; iii) emotional labour, and iv) taking distress home. The term ‘professional hierarchies’ refers to the structural dimensions of social relationships between people employed at various levels in organisations such as hospitals and other healthcare institutions. These aspects were central in the JMOs’ accounts of workplace experiences; both positive and negative. Two professional relationships that were embedded within the training hierarchy and frequently mentioned by the JMOs were the relationship between supervisor and junior doctor, and the relationships between fellow junior doctors. Key sources of workplace stress were a highly demanding workplace and adverse working conditions, time demands and lack of control over shifts and rostering, high expectations of JMOs and the continual transitions JMOs navigate. Responses to workplace stress included a fear of making mistakes, insecurity, feeling ‘trapped’ in medicine and exhaustion. The third main theme is that of ‘emotional labour’: the demands on JMOs to conform to emotion rules that involved both providing care to others and expectations to control their feelings so as to be seen as behaving professionally and competently in the workplace. The fourth main and novel theme is ‘taking distress home’, which highlights how JMOs’ experiences of feeling overwhelmed and anxious knows no boundaries.

These themes are intertwined, so that professional hierarchies contribute to, or in some cases, mitigate workplace stress, while emotional labour is central to managing the stress created by non-supportive hierarchies. Stress permeates home and work and affects relations within these spaces. The transitional nature of the training program, with term-to-term placements and rotations through teams and specialties, creates a cyclic dynamic of upheaval and uncertainty. This exacerbates some of the anxiety-laden responses and accentuates the need for stable supportive professional mentorship and peer cohesion to engender a sense of belonging and shared understanding.

### Theme 1: professional hierarchies

Professional hierarchies were enacted through relationships between different levels of the hierarchical structure of medicine and the hospital. These levels included the administration, senior consultants and clinicians, supervisors throughout JMO placements, teams within wards, and fellow medical JMOs. Crucially, these relationships could be negative or positive in nature, and could impact on JMOs’ well-being for better or worse. Chief actors in these relationships were senior clinicians, supervisors of JMOs, colleagues and fellow JMOs, as well as the broader professional structure of medicine.

Negative professional hierarchies could be characterised by an imbalance of power, absence of personal connection or scarcity of availability. Several JMOs described unsupportive or distant senior doctors. Charlie noted, for example, that ‘*my clinical supervisor I haven’t seen in like a year, and lots of the consultants, you don’t really see them’*. Some described a power hierarchy between supervisors and JMOs, and instances of bullying and dismissive interactions from time-poor consultants who ‘*defended their time aggressively*’ (Sam). Within a stressful working environment, relational disconnection from colleagues generated a sense of de-valuation and low self-esteem for some JMOs: ‘*I felt like my bosses didn’t really value me, or my other registrars didn’t really value me in the workplace’* (Alex). There was a common theme of insecurity around professional competence and some JMOs expressed a fear of others’ negative evaluation. With most JMOs on one-year contracts, an additional recurrent source of stress was that of annual job applications, a process that for some JMOs, fostered this insecurity around professional competence.

Professional relationships could be positive and enhance JMO well-being if they were supportive, consistent, available, and dedicated to developing better doctors and delivering better healthcare. Supervisors occupied a position within the medical hierarchy that rendered them able to provide clinical advice, and professional and personal support to the JMOs. When supervisors fulfilled their support responsibilities, the performance and coping of junior doctors was constructively addressed. These relational connections promoted a sense of being valued, both as a person and a JMO doctor, and being respected as a learner. JMOs described different contexts and spaces in which supervisors could provide mentorship and support to JMOs both formally and informally. These examples included proactive supervisors undertaking regular check-ins and teaching on the wards, informal catch-ups and advice over on-call consultation at the bedside, where supervisors co-navigated the care of patients with JMOs, and in turn, showed care for their supervisees. Scheduling mid and end-of-placement check-ins and formalising regular mentorship times within training programs could set aside formal spaces for mentorship and supervision to occur more frequently.

The importance of developing this respect and feelings of greater confidence was evident in many accounts, where JMOs described a process of trust, learning, and the development of mastery and competence in clinical skills: *‘as you go on, you realise even those little mistakes*. *.. you can learn from them’* (Alex). For some JMOs, training generated an affective atmosphere of stimulation and intellectual challenge. They experienced this time as an opportunity to develop competence and mastery: *‘the best part of being a JMO was actually reinforcing all the things that you’ve learnt and putting them into practice on daily basis and feeling like you’re progressing as someone in the medical field’* (Robin). These findings demonstrate how a supportive supervisory relationship can help JMOs manage their fear of making mistakes and to learn how to deal with workplace stressors more effectively, and how opportunities for face-to-face teaching on the ward can positively influence JMO self-esteem and professional development.

Peer relationships and feelings of team belonging were important facilitators of JMO well-being. Many participants identified that a key source of support for a JMO was their fellow junior doctors. These were crucial relationships of shared experience and understanding that were largely positive and formed a consistent base for JMOs in the face of the regular transitions as they moved through their training program. Long hours at work meant that the majority of JMOs spent their time together in shared spaces as they participated in the training program together. In experiencing the same fears, stressors and dilemmas, the cohort of junior doctors provided shared understanding, common humanity and a sense of comradery that was evident in many spaces and formats. Many JMOs described instances of positive, plentiful support, and a sense of altruism and dedication to helping out fellow JMOs: *‘as an intern you know like, everyone wanted to work hard, be the best, go have coffee together and then help their mates if they finished their job early’* (Alex). This sense of team cohesion, of being valued and supported by colleagues, particularly fellow JMOs, was an important factor in generating motivation, professional identity and shared purpose.

### Theme 2: workplace stress

Several of our interviewees had previously worked in other professions, and one commented that medicine was ‘*the hardest job I’ve ever done*’ (Jamie). JMOs described working under intense time pressure and described cognitive and emotional strain from multiple demands on their attention. Adverse working conditions were described as a visceral source of stress for many JMOs. These included long hours, high patient loads, infrequent breaks, high job demands and low job control. There did appear to be individual differences between the participants in the degree to which they reported experiencing distressing affective responses to working as a JMO and in the coping strategies they adopted. Some JMOs reported that their affective responses can change over time as a result of experience and changes in location.

For many participants, being stretched in terms of time was a prominent source of stress that left them vulnerable and exhausted. When combined with a high patient load and understaffing, study and training demands contributed to a sense of endless pressure, over which JMOs had little control. Even the most basic requirements for their physical well-being were commonly lacking on hospital premises, such as no quiet spaces on the wards. Working without lunch breaks, and not being able to go to the bathroom, were commonly reported.

It was not only a matter of working hours (per se), but the lack of control the JMOs had over determining those hours, and of having too much to do within those rostered hours. More broadly, it was the lack of power over these aspects of their work that was described as most problematic for their mental health. Regardless of how hard they worked, JMOs were at the mercy of the organisational factors such as understaffing and unrealistic patient loads and carried their pagers at all times. Ultimately, top-down administrative pressures prevented JMOs from maintaining a healthy work-life balance during and after work hours. Procedural barriers and administrative difficulties in rostering were practical obstacles for some JMOs in applying for holiday or sick leave or attending appointments. Improvements in workplace staffing and shift scheduling would provide more human resources and material support to assist doctors.

Workplace stress associated with the external factors discussed above was at times exacerbated by internal factors, such as JMOs’ own interpretations, personality traits, self-stigma or internalised expectations of the medical profession. Insightfully, one JMO identified a process of normalisation of extremely high standards in medicine, where: *‘everyone has pretty high standards of themselves. .. And it’s hard because when you’re around that environment that becomes normal’* (Alex). This tendency was identified in some JMO accounts when describing themselves and their cohort as ‘*Type A medical people*’ (Alex). Upon reflection, Alex noted that regardless of one’s personality, the extraordinary demands of the job would make anyone stressed. The skewed baseline for comparison meant JMOs only compared their performance to a very high-achieving successful cohort of fellow peers. For some JMOs, this was a source of feelings of low worth.

The high stakes nature of the role, where *‘everything is a lot more serious because it’s people’s lives*’ (Jamie), generated a highly stressful affective atmosphere amongst JMOs. All participants reported a fear of making mistakes, particularly in the first intern year. For example, Alex described *‘thinking that everything, every mistake you could make could be potentially cataclysmic and result in death because, you know, you work in health in a hospital and you’re a doctor*’. Alex went on to observe that this fear was compounded by feelings of uncertainty, anxiety and unpreparedness: ***‘****that responsibility was somewhat frightening, and I felt like we weren’t really prepared that well in terms of everyday tasks’*.

This fear of making mistakes and feeling unprepared was exacerbated regularly due to the rotational nature of the JMO training program. Each term required placements at different locations and adaptation to new teams and new environments, accompanied by upheaval and self-doubt. This anxiety was exacerbated for some JMOs by a perceived lack of clinical support and advice when required, leaving them feeling unsupported, in what was described as a *‘sink or swim*’ training process. The relational connections for support and learning JMOs needed in these anxiety-provoking clinical situations were often not available or offered to them. Further, the wider context of medical practice generated extra anxiety for most JMOs. Anxiety about clinical decisions was exacerbated by the broader litigious culture of medicine in which doctors: ‘*practice really defensively because you’re aware that the current climate we’re in is quite sort of litigious’* (Charlie).

Several JMOs pointed to the gap between their expectations of training and the reality of living these experiences. One participant observed that JMOs were most vulnerable to mental and physical ill-health when there was a pronounced mismatch between their expectations and demands at and outside work. Relatedly, participants referred to the relentless training and career progression of a doctor as offering little opportunity to reflect realistically and self-compassionately on whether they were suited to the profession, and if it was sustainable for long-term well-being. Constant focus on study and examinations was required for career progression and entry into competitive specialist training programs. After lengthy study and intense sacrifice to the demands of work and exams, some JMOs described feeling trapped: ‘*lots of people feel that they can’t leave and it would be like a failure if they left medicine, even if they’re hating it and not well suited to it’* (Charlie). For some, leaving the training pathway from clinical medicine was considered ‘*unachievable*’ (Charlie). Education on career alternatives was identified by some participants as a necessity during training.

Interviewees felt that their time was devalued by management via administrative errors around leave and payment and inflexibility around rostering, and through procedural barriers and administrative difficulties that they encountered when applying for leave, particularly sick leave. These obstacles were further complicated by stigmatised attitudes towards illness within the workplace culture: *‘The logistics [were a problem] because then I would have to reveal that I was going to a medical appointment or a psychologist’s appointment’* (Jamie). Similarly, workplace stressors of understaffing and under-resourcing created a culture of guilt around taking leave, which was viewed as harmful to the team.

### Theme 3: emotional labour

Several prominent forms/acts of emotion rules and related emotional labour emerged from the interviews, which had largely harmful impacts. The tension between an expectation for outward displays of clinical competence and confidence, despite lack of experience, was a key source of anxiety that was recurrent throughout training stages. For example, Alex observed a cycle of waves of anxiety throughout their training. These feelings emerged at the commencement of a new hospital, a new term/speciality, in a new team, or annually at the commencement of a new role, and were continually recurring due to the rotating nature of their training and identity formation from medical student to competent doctor. Alex described: ‘*this spike of apprehension again which is a bit less so than before, but you suddenly realise okay, well now everybody expects me to perform at this next level and what if I don’t perform at this next level?. .. when people expect you to like have this whole new year’s worth of experience.’* (Alex). Given the daily experience of anxiety and distress, it was concerning that a number of JMOs expressed self-stigma and shame around their mental health because the emotion rules of their workplace demanded that medical staff should not display these feelings. For some, this was another barrier to help-seeking. Charlie described feeling *‘embarrassed to go to anyone about my work anxieties*’, even a GP. Robin reflected that seeking professional support ‘*would be a pretty big step for me to take. And. .. it would take, I think, a fair bit of nudging from a friend or a family member to get me to go there*’. Several participants reported that they did not have a regular GP. Charlie’s observation that *‘I feel like anxiety’s much easier to hide and manage*’, suggests an underlying inherent pressure for JMOs to hide their mental distress. An underlying stigma was also hinted at in some accounts, where a mask of humour, ‘*camaraderie and banter’* (Pat), was employed as a coping strategy to broach serious issues such as mental health problems within peer cohorts with a sense of distance and detachment: ‘*Like jokingly say, “I want to kill myself.” Things like that. Or, just like in a joking manner say, like, “If this happens, I’m going to shoot myself*”’ (Pat).

These sentiments and behaviours among JMOs, and among senior doctors, could be interpreted as a response to the enduring professional and cultural norms and expectations of a doctor as ‘invincible’, a provider of care to others, always placing patients first. Some participants reported feeling a sense of failure or weakness in the face of the silent expectation of a doctors’ ‘invincibility’, an idea also expressed by Mexican physicians-in-training [23]. In these Australian accounts there was a tendency for JMOs to downplay their worth as patients, reflecting that this was a barrier to help-seeking. One participant reflected: ‘*it’s not really like confidentiality. It’s more, and I guess like, the judgment from those health care professionals. We’re all dealing with that every day. You can manage this yourself. You’re kind of wasting their time.’* (Charlie).

Professional stigma was at times modelled by senior colleagues, with some JMOs describing a reluctance to openly discuss mental health issues within the workplace—for example extremely dismissive interactions with managerial staff around mental health sick leave and a failure to check-in regularly with JMOs about basic well-being. Additionally, the insularity of the ‘medical’ world also interfered with help-seeking via professional pathways. A lack of confidentiality within the profession was described as a barrier to JMOs accessing medical treatment, and for some, contributed to a sense of isolation and an unmet need for shared understanding. Furthermore, the closed nature of the profession meant that often a supervisor or senior could also be the individual tasked with deciding entry into specialty training programs. This situation complicated disclosure of mental health issues to senior staff.

To address these issues, Jamie suggested that JMOs require *‘a confidential sort of means for JMOs to talk to someone who sort of understood what they were going through*’. When help was sought, JMOs described a preference to seek help outside the local hospital system in which they worked to maintain confidentiality. A confidential specialist healthcare service for doctors, run by doctors, with appointments available that suit doctors’ rosters was called for, as was allocated time in rosters for appointments and check-ups that could be applied for easily and confidentially. A dedicated, independent medical service for doctors and streamlining the administrative processes for leave for appointments were suggested by JMOs. These changes would provide the material space and the organisational sanctioning for JMOs to receive medical or psychological care themselves.

One participant highlighted that deep systemic changes were required at an organisational and policy level to change the impact of workplace culture towards staff mental health. Expectations of ‘invincibility’ and structural and practical impediments to help-seeking and to a healthy work-life balance, generated a sense among some JMOs that their own needs were being de-valued by the organisation and the profession. These affects were perpetuated by the daily realities of excessive workloads and understaffing. Normalisation of these workplace demands, and of a devaluation of the participants’ basic needs and a lack of agency to assert these within the structures of administration and hierarchy (such as taking meal breaks), generated a collective silence among many JMOs. Some JMOs felt unable to voice their experiences with peers, other colleagues or supervisors, whilst others turned to other JMOs for understanding and support. There was anger expressed due to the perception that JMOs were being blamed for a lack of resilience, with one participant describing initiatives to improve resilience amongst JMOs as ‘*tokenistic offering [s]’* (Robin). One JMO stated: *‘yes, I kind of feel there needs to be some accountability from people higher up because there was a lot of focus on the JMOs being more resilient and it’s kind of insulting’* (Jamie).

### Theme 4: taking distress home

Critically, these distressing feelings were not confined to working hours or to hospital grounds. Some JMOs described an affective atmosphere of worry and rumination that continued at home, after hours. One JMO described this feeling as physiological arousal akin to them being on the ward in a time of critical medical context. Charlie noted that: ‘*I feel like you’re stressed for 12 hours that you’re at home about what the result was and what’s happened’* and described the resulting physiological hyperarousal at home as ‘*not being able to switch off, like you’re [still] at work*’. In this situation, JMOs have no space in which they can feel they can escape the pressures of work, and therefore, no chance for relief from these feelings even when they are off-site. Long working hours was a huge problem for some JMOs, resulting in sleep deprivation, isolation from family and friends, work-life imbalance and a limited capacity for healthy behaviours. Time spent in recreation or rest was minimal. The only space and pursuit in which JMOs were present was work-related. JMOs described temporal and physical dedication to being on site for extremely long working hours. They described a life phase in which previous strategies to promote well-being, such as socialising and exercise, were frequently abandoned to meet the demands of overtime and workload.

Many JMO accounts revealed a split or distinction, between ‘medical’ and ‘non-medical’ people and social relationships. This could be observed in the accounts, for example, in explicitly identifying a partner as ‘non-medical’ or implicitly referring to a collective ‘us’ when mentioning the JMO cohort. Their position within or outside this context, engenders a distinct professional, social and personal identity for the JMO themselves, and their relationships. On a practical level, long hours and overtime prevented JMOs from fostering non-medical connections and spending time with family and friends or on holiday. Some JMOs described feeling distant emotionally, physically and temporally from their friends and family, of having no common ground, spending no downtime at home after all day at work. Overtime and shift work upended routines of sleep, meal times and weekends. Many JMOs expressed feeling torn and sometimes guilt-ridden as they negotiated the tension between the demands of the medical world, and their non-medical interests and connections. One participant described their feelings using the metaphor of emptiness and a hollowing out of selfhood: ‘*I felt like I was burning-out, and a bit of a shell of myself*’ (Alex).

Separation and isolation were also evident at a relational level. Those ‘non-medical’ individuals, such as family or friends who do not have awareness of the affective realities of the medical world, of the daily exhaustion and pressure of the hospital, were viewed by the participants as not connected to the JMOs’ experiences. The implicit shared understanding that being a fellow ‘medic’ brings, and the lack of understanding with those outside the ‘medical’ sphere, can either open or close relational connections and opportunities for support and validation for JMOs amongst their social networks. Alex described feeling not understood by friends and family, and an inability to foster such understanding in these relationships. This generated an affective atmosphere of loneliness and social isolation outside of work.

## Discussion

Our study has highlighted the multifaceted dimensions of distress as well as the conditions under which JMOs can find support and identified their ideas for how to ameliorate the working environment that offers so many challenges to their well-being. We identified the different roles that colleagues, teams, supervisors, cultural expectations and norms, temporal, organisational and structural forces all have in shaping the experiences of JMOs, and the impact these factors have on their mental health and professional development. The analysis acknowledges the importance of relational connections and the workplace environment in shaping JMOs’ social and emotional well-being. Similar to previous research, our study identified a number of workplace factors and challenges to JMOs’ mental health [3, 5, 8, 24–26]. The findings highlight the relational and structural aspects to these factors to contextualise and dynamically situate these workplace conditions and stressors and understand their affective resonances for JMOs. The findings highlighted that distress and stress are most evident when tensions occur within and between the environment, resources and experiences of the JMO. When such tensions are incompatible or left unresolved, overwhelming stress, pressure and fatigue are generated in the JMOs’ experiences and environment. Participants’ accounts reveal a consistent affective atmosphere of anxiety, stress and demanding work environment, involving continual tensions. These include tensions between meeting the needs of the doctor versus those of their patients, colleagues, supervisors and the organisation and tensions between performing in a high-stakes, highly demanding environment with little safety net provided from their supervisors when mistakes are made. Disparities between expectations directed towards JMOs versus their competence as still training doctors and between the need for JMOs to demonstrate competence despite a lack of on-the-job support to achieve this also created tensions for JMOs. Finally, the incompatibility between the ‘medical’ lifeworld of the JMOs versus the ‘non-medical’ world generated tensions for JMOs in relation to their life outside the hospital, at times resulting in feelings of disconnection from ‘non-medical’ networks.

Workplace stress has been associated with poor employee mental health [27], including medical practitioners [1]. Our qualitative findings update this literature by elaborating on contemporary sources of workplace stress for JMOs in the Australian health system context. Interviewees revealed that both the culture of medicine and the specific workplace stressors they were experiencing together engineered a devaluation of self. Many reported being overworked, under-valued and over-burdened across a number of spheres – cognitive, emotional, physical and academic. Similar stressors have been identified in qualitative studies with junior doctors in Australia and overseas [8, 24–26]. Lack of control over their working hours and conditions meant that many JMOs were institutionally placed to believe that they were not worthy of having a life outside medicine and their needs were inferior to those of the hospital. JMOs were also called on to engage in intense management and control of their emotions as part of presenting a professional demeanour and meeting training demands, which has also been noted internationally [15]. These findings are in line with previous research that has highlighted the deep-seated nature of these long-standing professional cultural norms and expectations within medicine, particularly those embedded in systems of authority [28].

The findings emphasised the importance of care: not just the demands of caregiving as expressed by the JMOs but their own need to feel cared for by supervisors, colleagues, friends and family so that the emotional demands of their workplace could be alleviated. We focused on how JMOs described feeling safe, secure, and comfortable or threatened, at-risk, and stressed, as a result of encounters with different factors including; i) other staff members, patients, and colleagues in professional hierarchies, ii) demands of training programs, iii) larger systems of administrative processes and structural hierarchies within the organisation, and iii) the medical culture itself. Results emphasise the role of training programs and workplaces in allowing for work-life balance for JMOs and the need for adequate leave, healthy working hours, improved staffing and rostering practices. These practices are also necessary for mental health to offset the highly stressful workplace and harmful working conditions JMOs face daily. A sacrifice of personal for the professional was evident across accounts, working overtime and long hours to deal with excessive workloads, leaving no time for rest or recreation. This result situates re-engagement with hobbies and close relationships with family and friends as a potential setting in which JMOs can be cared for and establish personal agency. The study identifies a novel additional pathway of support for JMOs beyond seniors and peers in the ‘medical’ workplace to friends and family in the ‘non-medical’ world.

There is a critical need to recognise and value emotional labour and ensure that support and supervision is in place to allow JMOs to cope with work demands [18] [19]. Our findings engage in new ways with concepts raised in Hochschild’s [17] work and recent research on emotion rules and emotional labour in health care [18–20], and extend these to novel domains of Australian medical training and the JMO experience. Our study highlights the need to validate the JMOs’ own struggles with distress, to provide them with workplaces that enable JMOs to activate their own capacities for self-care and to assist them in seeking external help from professional or personal supports. Furthermore, the accounts emphasised that such distress needs to be met with compassion, not blame for a perceived lack of resilience or stigma. Instead, mental ill-health needs to be understood as stemming from a combination of individual, work-related and systemic factors [4, 27]. Indeed, there is clear evidence that doctors do not suffer from a deficit in resilience [29] and that a greater focus on organisational strategies to improve doctors mental health is urgently needed [30, 31].

Our findings highlight that organisational and supervisory structures for JMO training and support are often lacking and require improvements through policy and processes to provide support for JMOs. Supervisors and senior doctors were key relationships for JMOs well-being, educational and professional development, as identified in previous qualitative studies [24]. These relationships were described as positive or negative and could impact JMO well-being for better or worse. The issue of lack of confidence, trust and support between senior and junior doctors was raised by some JMOs. There are many reasons for this, some of which were raised in the accounts, such as senior colleagues being time-poor, the structural power imbalance embedded in the training programs, JMOs hearing or observing instances where their peers are treated negatively when they raise mental health problems, and concerns around professional stigma towards mental illness [32], confidentiality and mandatory reporting. Additionally, senior clinicians are usually the gatekeepers of their future career, for example, in charge of decisions on entry to competitive fellowship programs, so it is not surprising that fear of ramifications for career progression is another barrier for JMOs to disclose mental health issues to their senior counterparts, as noted previously [12, 33]. There needs to be systemic change in the medical culture and within training programs so that JMOs can speak openly to senior colleagues, can receive timely help and improve their well-being, without negative impacts on their future careers. Ways to provide JMOs with more support through these supervisory relationships could include formal and informal strategies, some of which were suggested within the interviews. These could include regular check-ins with senior staff at rounds and breaks, mid/end-of-term placement feedback and protected regular mentorship sessions with an independent mentor who follows students throughout their training. Another promising strategy is manager mental health training [34], where managers are trained and upskilled to identify and respond effectively to mental ill-health in their direct reports. This approach has been used with other at-risk workforces such as emergency services and has seen positive results for manager and employee outcomes [35, 36]. Mental health training for physician supervisors is currently being piloted with senior doctors in Australia [37, 38].

The role of generational and professional cultural norms within medicine also needs to be recognised when considering these findings. Whilst we acknowledge that long-standing attitudes and norms are not easily or quickly addressed, a promising strategy to promote a more positive workplace culture is to lead cultural change from the top-down and again, manager mental health training may be a way to foster this change. Equipping senior managers with skills and behaviours to identify and respond effectively to employee’s mental health problems can provide positive role modelling, encourage future disclosure and facilitate help-seeking throughout an organisation [34]. Further research is warranted regarding strategies to provide JMOs with more support throughout their medical training, such as supervisor training.

More broadly, our results clearly emphasise that JMO mental health needs to be understood within a multi-level framework that acknowledges the multifactorial nature of employee well-being [39]. Drawing on best-practice evidence to date, this framework recommends that a coordinated range of initiatives is implemented simultaneously at the individual, team, organisational, and in the case of medicine, cultural and professional levels, and the health system more broadly [39, 40]. Clearly, the unique and complex context of medical training will require that strategies be modified for doctors [41], and a tailored suite of interventions in this space has been proposed in a recent review of doctors mental health [42]. Internationally, medical bodies and reports have also called for a multi-layered approach to address doctors’ well-being [43, 44]. However, in-depth qualitative research such as this and similar studies [45] are an essential first step in this process because they provide an in-depth first-hand exploration of local work-related factors that affect JMO mental health. Our study clearly indicates the need to provide JMOs with workplace policies, teams and a professional culture that are conducive to employee well-being. This aligns with recent calls from key professional bodies for a greater focus on doctors mental health within professional training programs [46, 47]. Our findings suggest that efforts to this end will require changes not only to medical colleges and training programs, but also to workplace processes and policies, organisational support structures, and medical culture .

The findings contribute to and extend previous work by delving into the complexities and emergent nature of contemporary Australian JMOs’ affective experiences in context, including the relational connections they experienced in the workplace and how these sat within the professional hierarchy of medicine. Consistent across all accounts were the continual transitions between social, structural and affective contexts that JMOs had to navigate each term which required huge sacrifices and resulted in major work-life-well-being imbalances. Transitions between terms, different placements, hospitals, team and supervisor groupings, between work and home, and between ‘medical’ and ‘non-medical’ domains were continuous. It is notable from our interviews that despite decades of research into the reasons for poor mental health among junior doctors, for these JMOs, little seems to have changed in terms of their workplace, either structurally or practically, its influence on their well-being, nor in the institution’s progress in addressing these problems of hierarchy and systemic stigma in response to mental ill-health and dealing with JMOs’ challenging working conditions. What is clear in this study is that the JMOs felt that hospital management and the general institutional structures not only demonstrated a lack of care for their health and well-being but actively undermined it in ways that hampered their own capacity to engage in self-care and the care of colleagues and patients.

The potential for professional hierarchies to be either supportive or unsupportive, and to have both positive and negative impacts was highlighted in our findings. As JMOs, workplace relations were often embedded within and built upon entrenched power hierarchies and professional norms within medicine, as has been noted internationally [15]. The accounts emphasised that supervision was a key relational connection that could be harnessed to improve both the training and support provided to junior doctors. For example, the quality of the learning environment has been shown to be a significant risk factor for burnout in Dutch residents [48]. Work teams and peer cohort were also described as potential sources of support, promising because these relational connections are already embedded in the structural hierarchy of the workplace and provide an accessible avenue for positive mentorship and support, whether formal or informal.

In summary, the study delves deeply into four intertwined core dimensions of JMOs’ experiences that have not yet been explored in the contemporary Australian health system context and is therefore able to highlight the continuities across medical training worldwide, whilst providing unique insights from Australian experiences.

The study adds to previous literature most notably in three ways: i) it extends its focus from the care offered to patients to the conditions under which JMOs offer care and require it themselves; ii) it addresses relationships and other social dimensions both within the workplace and outside; and, iii) it offers an affirmative perspective by identifying not only the factors contributing to poor mental health and but also those that can support JMOs’ health and well-being. The analysis highlights the capacities within each junior doctor for self-care and to care for patients, and the capacity for peers, team, supervisors, training programs and support networks outside medicine, encouraging a multi-level approach to future strategies for supporting well-being. These findings have important implications to inform new strategies to improve JMO mental health and to leverage work and non-work contexts to better support JMOs during medical training.

A number of study limitations require consideration. Recruitment was voluntary and may reflect self-selection bias. However, given that doctors’ response rates are typically low [49], we felt that this was necessary to engage sufficient sample size from a population who are on frequent workplace rotations and time-poor. The participant group for our study was recruited from large metropolitan hospitals and as such, their workplace experiences may not reflect those of junior doctors in non-metropolitan or smaller hospitals. While the sample included a range of genders and both under- and postgraduate educational pathways, the junior doctors participating in this study were predominantly of English-speaking background, and all but one had trained in metropolitan Australian cities. Future research could include a more diverse sample including individuals born and/or trained overseas, and from a range of socioeconomic and demographic backgrounds.

## Conclusion

Recent models acknowledge that interventions for employee mental health must address all levels simultaneously; individual, team, organisational and systemic [39, 40] and must implement strategies that consider the specificities of the workplace context. Insights from our analysis can inform the design of tailored interventions that account for the emotional, organisational and relational dimensions of JMOs’ experience. In attending to the tensions, stressors and transitions that JMOs constantly encounter within medical training, this study suggests new ways to promote the mental health of junior doctors. These findings will inform the development of interventions and changes in practice within organisations, teams, training programs and workplaces.

## Supplementary Information

**Additional file 1.** Interview Topic Guide.

## Data Availability

Data generated and analysed during this study contain sensitive personal data and will not be made publicly available to maintain the privacy and confidentiality of our participants.
