# Supplementary material for: ‘The hardest job I’ve ever done’: a qualitative exploration of the factors affecting junior doctors’ mental health and well-being during medical training in Australia
Source: BMC Health Serv Res. 2021 Dec 14;21:1342. doi: 10.1186/s12913-021-07381-5 (PMC8672528; doi:10.1186/s12913-021-07381-5)
Supplement: Supplementary file 1 — Additional file 1. Interview Topic Guide. [file 12913_2021_7381_MOESM1_ESM.docx]

**Additional file 1:** Interview Topic Guide

**Part 1: Mental health and wellbeing as a JMO**

**JMO life and mental health**

Can you tell me a little bit about your experience being a JMO?

PROMPTS:

- hardest thing about being a JMO?
- best thing / most rewarding aspect of being a JMO?

What do you think are the main stressors (sources of stress) as a JMO?

What are the three main workplace stressors?

What are the three main non-workplace stressors?

PROMPT: not enough time to… (spend time with partner/family, exercise etc)

What are the toughest periods of your JMO career and training? (when/timing)

Has it been harder or easier being a JMO than you expected?

PROMPTS:

- times of transition? i.e. hospital-to-hospital (limited inductions, unhelpful administrative staff)
- stage to stage? i.e. beginning to supervise others/- examinations, applying for program entry

How have you found working in a regional hospital?

What would make working in a regional area easier? Do you have any tips for JMOs working in regional areas?

How confident are you in recognising symptoms of common mental illnesses, such as depression, anxiety or PTSD?

What role do you think sleep (or lack thereof) plays in the mental health of JMOs?

What things do you think could help prevent mental health problems amongst JMOs?

Are there techniques or skills that you think JMOs should learn to help keep well?

**Help-seeking (Barriers? Facilitators? Sources? When?)**

If you were to experience symptoms of a mental illness, would you feel comfortable seeking help for these problems?

PROMPTS:

- If yes, why? What contributes to this? (facilitators)
- If no, why? What are the major barriers?
- What might make other JMOs reluctant about seeking help for mental health problems?

If you were to experience symptoms of mental illness, where would you go to seek help?

PROMPTS:

- professional
- Online or app-based treatment

At what stage / when would you seek help?

PROMPT:

- early on, initial symptoms, before it affected their work;
- only when it begun to affect professional actions or quality of care?
- ignore it till crisis point?

Are you aware of any services or initiatives to support the mental health of JMOs?

Do you think you would use a smartphone app or online services to help if you were experiencing problems?

After a quick break, we will move on to discuss the use of apps.

**Part 2: Technology and apps for mental health – in general & for JMO’S mental health**

**Technology and app use in general**

Are you using any apps on your smartphone at the moment, and if so, which ones? How frequently?

- What types? (e.g. communication/messaging; health-related; daily life planning)
- What is your go-to app you open everyday?

What apps, if any, do you use in the workplace?

What apps, if any, do you use outside of the workplace?

What do you like about them? (draw-cards)

What do you not like about them? (negative aspects, drawbacks)

What are your major concerns around apps on your smartphone? (e.g. privacy, security, cost)

What are the main advantages that apps offer?

Can you access free wifi in the hospital?

**An app for JMO mental health**

What are your thoughts about using an app for supporting the mental health and wellbeing of JMOS?

PROMPTS

- What for / how would you use it? If not, why not?

What are your thoughts about using such an app to prevent future mental health problems JMOs?/using such an app to help support JMOs with current mental health problems?

When / how often / for how long do you think you would use an app for JMO mental health?

What would be your bare minimum available time in a day or week during which you would realistically have to spare to use an app for mental health?

**Privacy**

What identifying information would you feel comfortable entering into a Black Dog Institute app for the mental health of JMOs? (eg full name, telephone number, address, email account, current role and workplace)

**App Content and Features**

What types of therapeutic content would you like, or not like, to see?

PROMPT: Mindfulness, CBT, BAT, Sleep, mood monitoring

What features would you like to see offered in an app for JMOs?

PROMPT: What do they NOT want? What would they resist?

What topics / life areas / stressors need to be addressed in an app for JMO’s?

PROMPT: sleep, stress management, work/life balance

If you were writing up a wish list for preferences / must-haves for a JMO mental health app, what would be at the top 3 of your list?

What would motivate you to continue using this app?

What might stop you from using a mental health app for JMOs?

Can you work around / modify any of these barriers?

**Name**

What name would you suggest for an app focused on the mental health of JMOs?

Can you describe one key example for you that captures this issue of ‘mental health’?

e.g. could be an image, a quote, a person/character? from the media, the arts (a book , a film, a TV series, a material object)

**Concluding Check-In**
